# The Story of the Insane from Year to Year

**Published:** 1904-07-16

**Authors:** 


### THE STORY OF THE INSANE FROM YEAR TO YEAR.

### SIXTEENTH ANNUAL SERIES.

#### EARLSWOOD ASYLUM FOR IDIOTS.

ON the first of January this asylum contained 513 inmates, and at the close of the year the number had increased to 522. There were 47 first admissions, 7 readmissions, 34 were discharged relieved, and 13 died. Table II. shows the assigned causes of the idiocy or imbecility in the year's admissions, and from it we learn that 23 cases were hereditary, and 2 owing to consanguineous marriages. "Maternal shock or fright" is supposed to have been the cause in 16 cases. Epilepsy and other causes account for 30, and in 12 no cause was known. Of the 13 deaths 5 were due to consumption. New rules have been framed for the management of the institution. The Local Government Board having undertaken the education and training of children who can fairly be paid for out of the rates, it follows that such places as Earlswood will become schools for the children of those who cannot provide the expense of purely private asylums and who are above the rate-supported class. It is these intermediates whose cases are often very hard, and Earlswood will be doing really good work by devoting itself to the relief of such cases; but it must jealously guard the sums to be paid, which should be as low as possible, and it should never be on the look-out for patients who can pay high rates.

